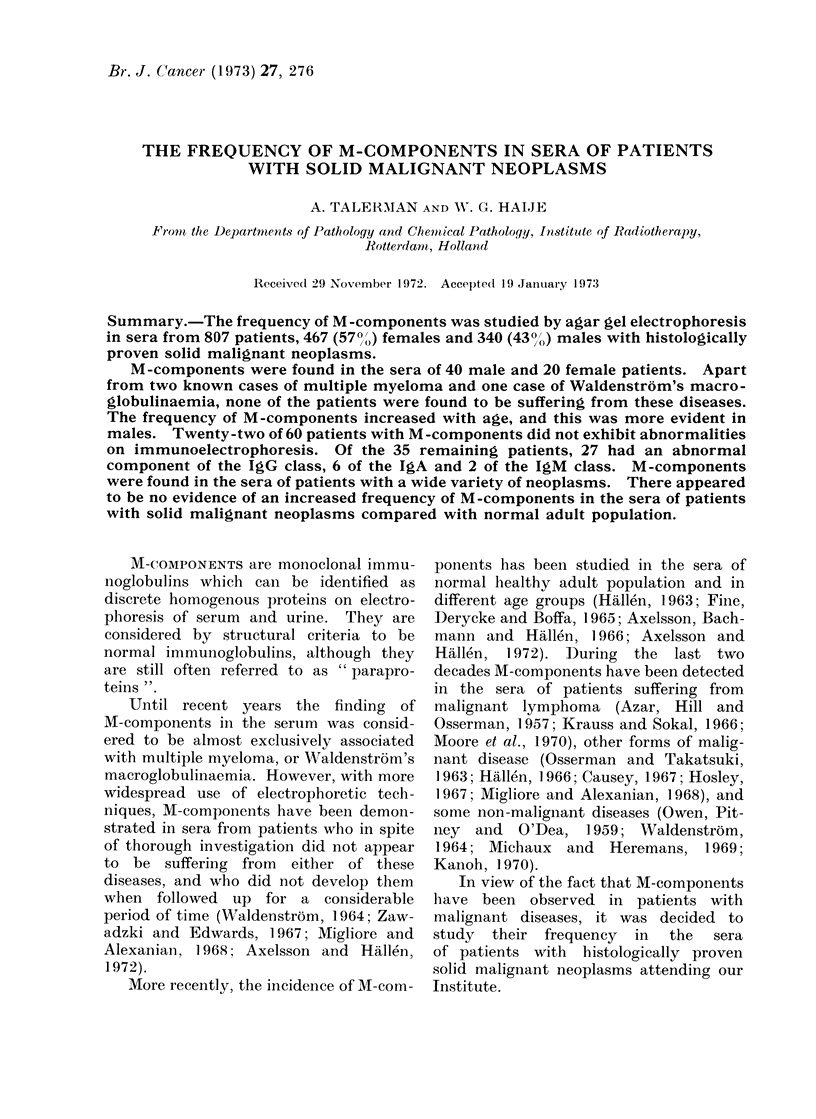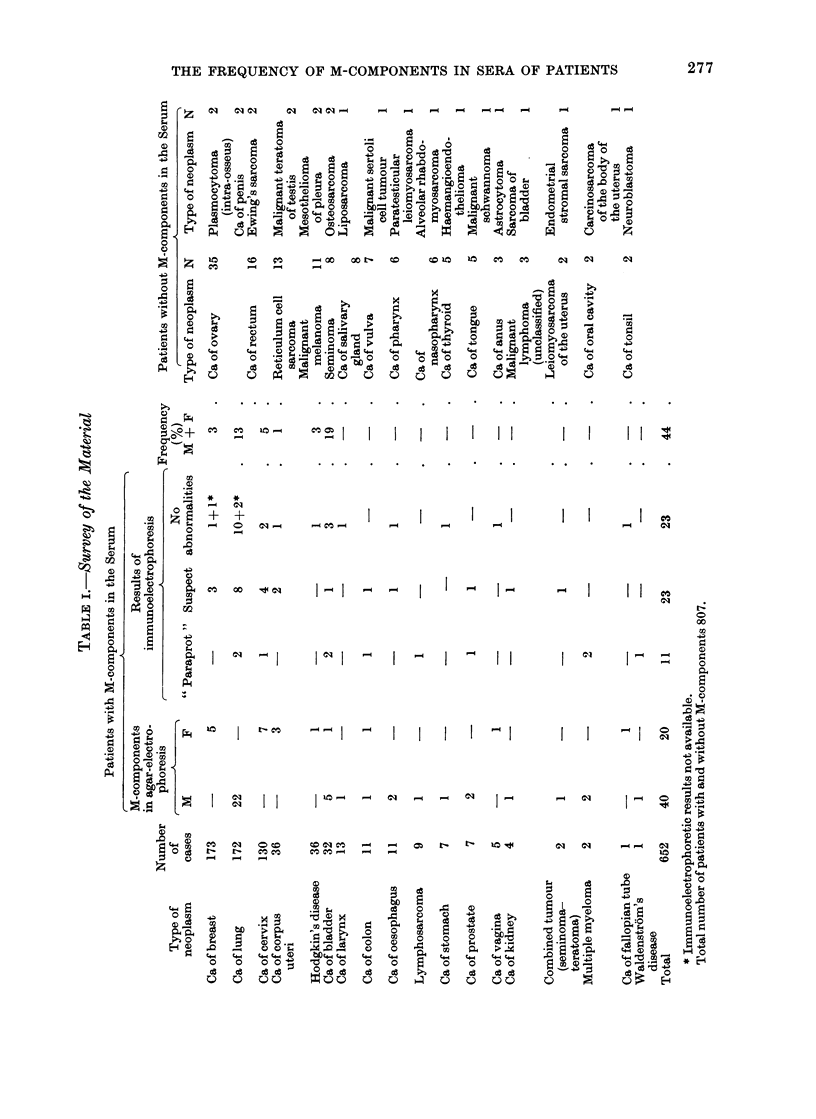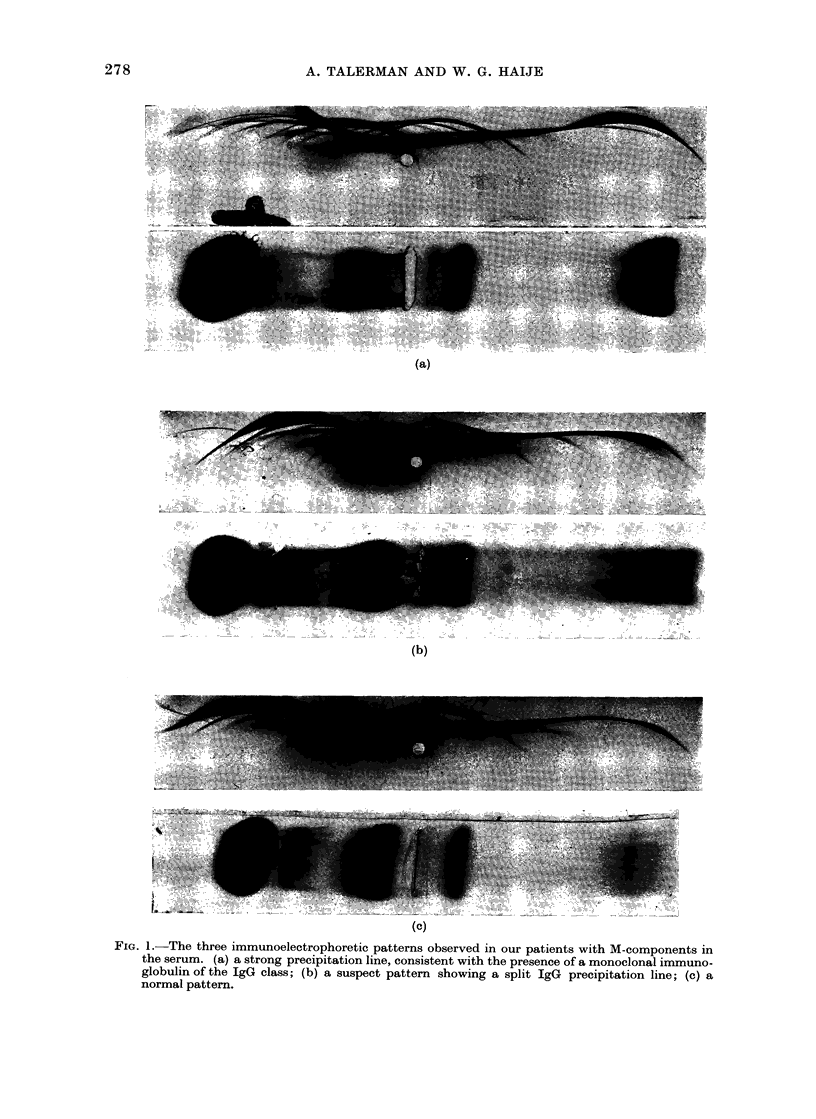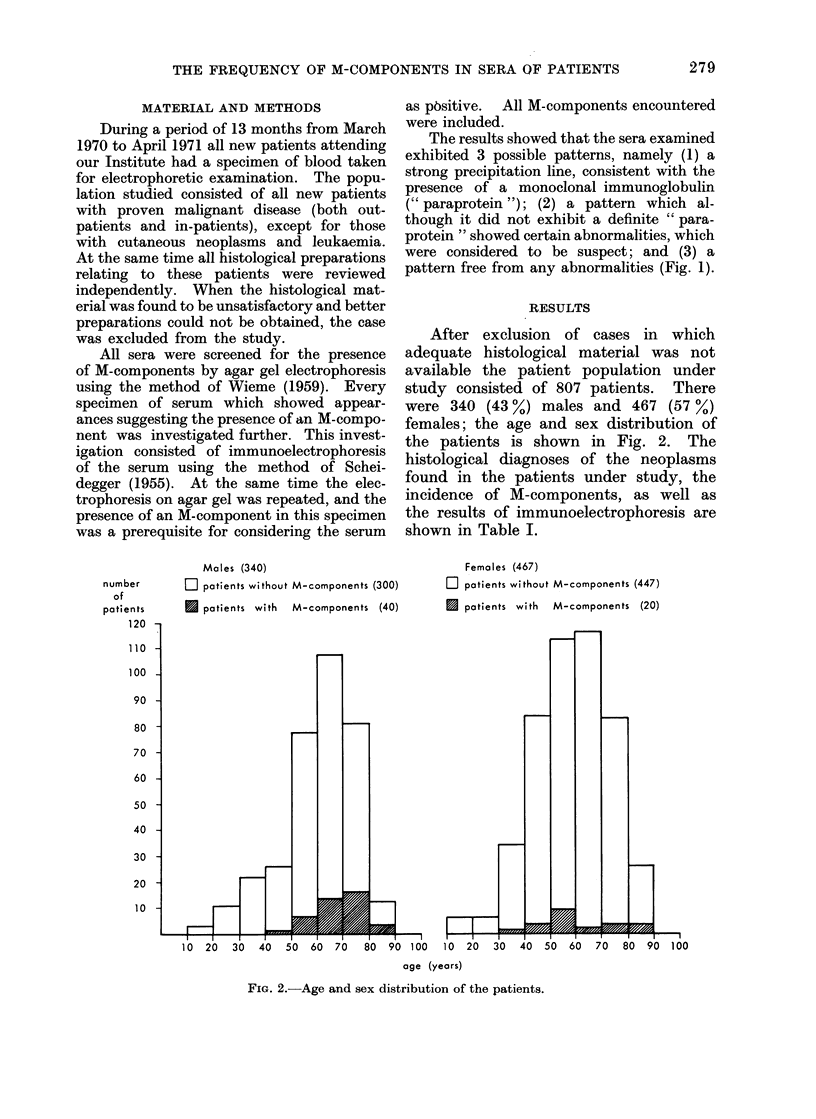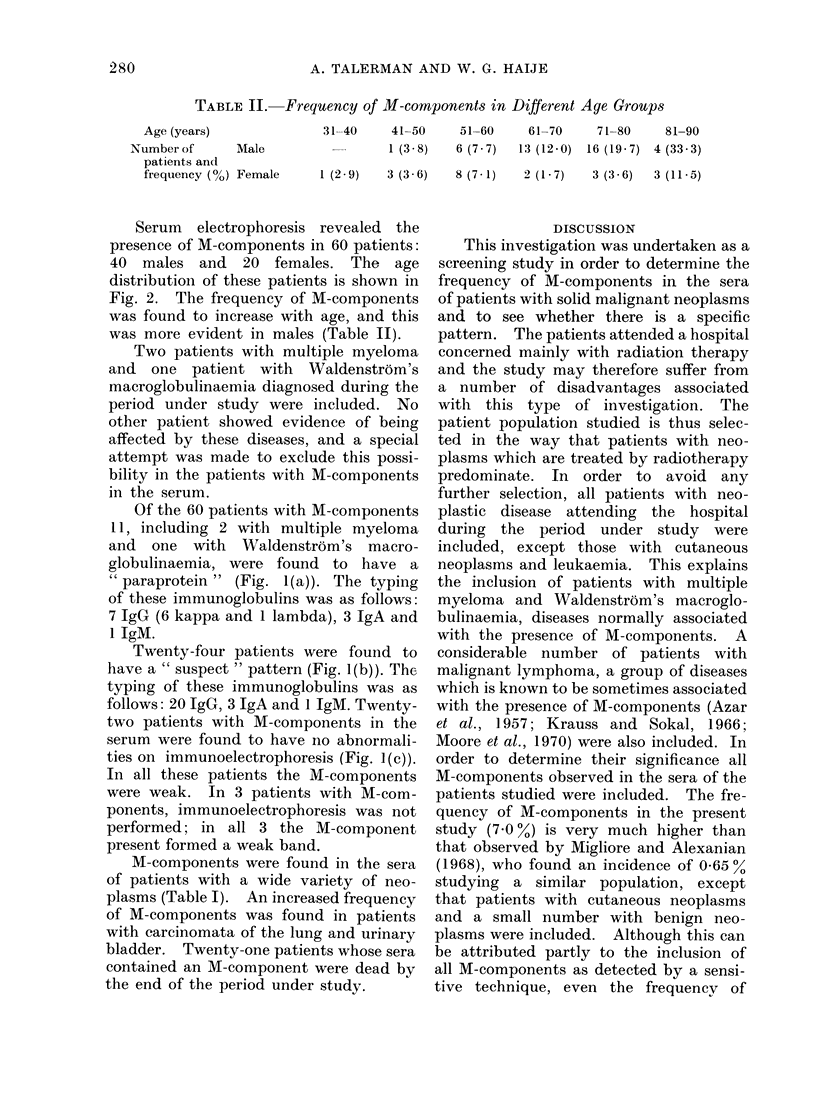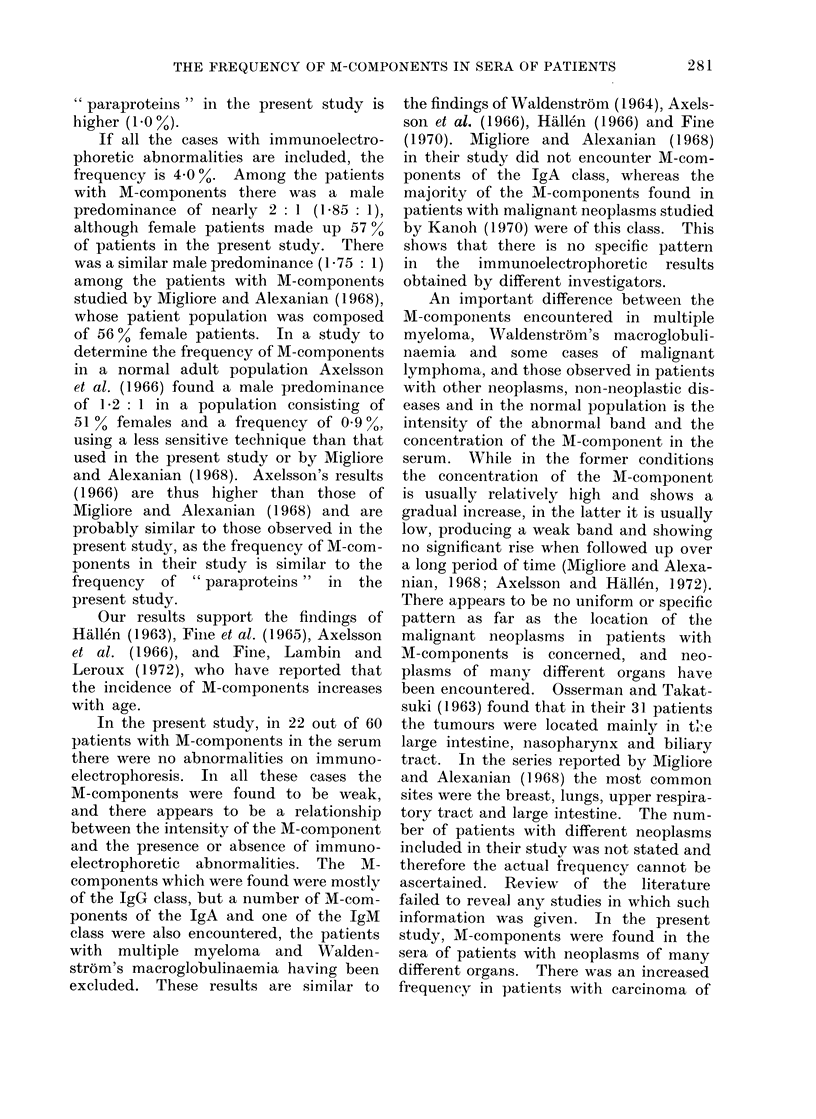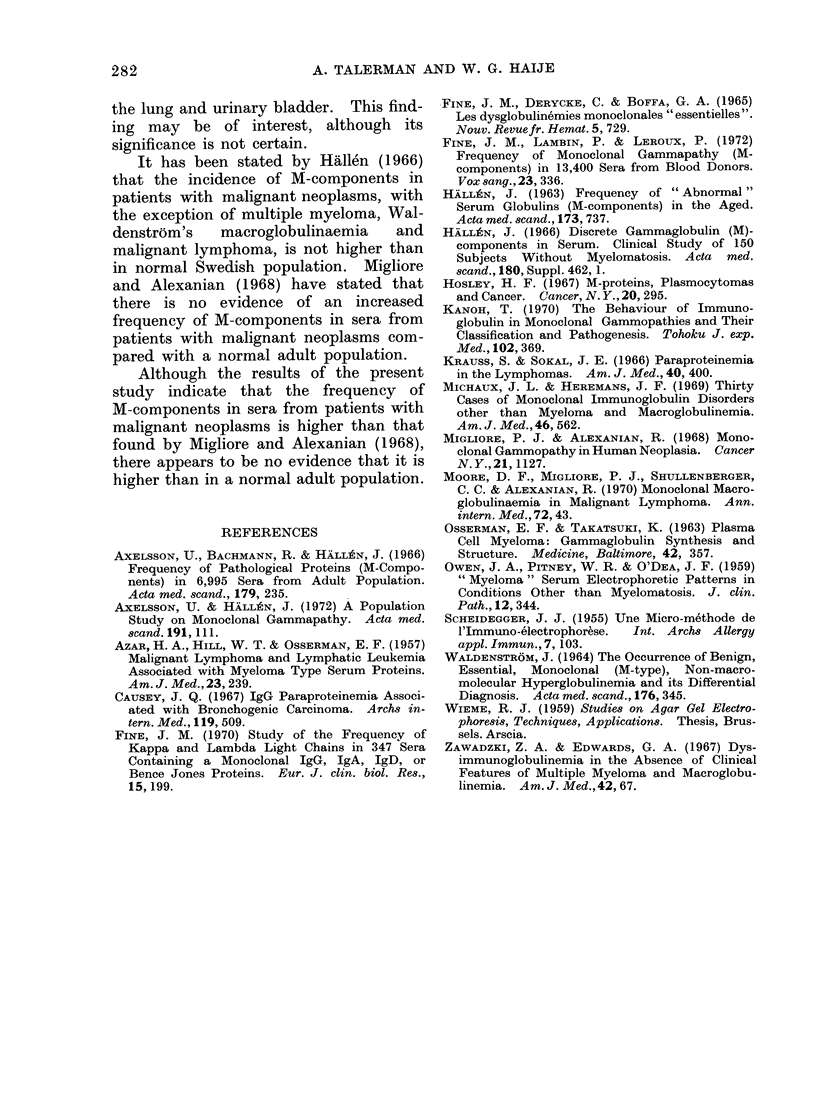# The Frequency of M-components in Sera of Patients with Solid Malignant Neoplasms

**DOI:** 10.1038/bjc.1973.34

**Published:** 1973-04

**Authors:** A. Talerman, W. G. Haije

## Abstract

**Images:**


*Br. J. Cancer* (1973) **27**, 276

### THE FREQUENCY OF M-COMPONENTS IN SERA OF PATIENTS WITH SOLID MALIGNANT NEOPLASMS

A. TALERMAN AND W. G. HAIJE

*From the Departments of Pathology and Chemical Pathology, Institute of Radiotherapy, Rotterdam, Holland*

Received 29 November 1972. Accepted 19 January 1973

**Summary.—The frequency of M-components was studied by agar gel electrophoresis in sera from 807 patients, 467 (57%) females and 340 (43%) males with histologically proven solid malignant neoplasms.**

**M-components were found in the sera of 40 male and 20 female patients. Apart from two known cases of multiple myeloma and one case of Waldenström's macroglobulinaemia, none of the patients were found to be suffering from these diseases. The frequency of M-components increased with age, and this was more evident in males. Twenty-two of 60 patients with M-components did not exhibit abnormalities on immunoelectrophoresis. Of the 35 remaining patients, 27 had an abnormal component of the IgG class, 6 of the IgA and 2 of the IgM class. M-components were found in the sera of patients with a wide variety of neoplasms. There appeared to be no evidence of an increased frequency of M-components in the sera of patients with solid malignant neoplasms compared with normal adult population.**

M-COMPONENTS are monoclonal immunoglobulins which can be identified as discrete homogenous proteins on electrophoresis of serum and urine. They are considered by structural criteria to be normal immunoglobulins, although they are still often referred to as " paraproteins ".

Until recent years the finding of M-components in the serum was considered to be almost exclusively associated with multiple myeloma, or Waldenström's macroglobulinaemia. However, with more widespread use of electrophoretic techniques, M-components have been demonstrated in sera from patients who in spite of thorough investigation did not appear to be suffering from either of these diseases, and who did not develop them when followed up for a considerable period of time (Waldenström, 1964; Zawadzki and Edwards, 1967; Migliore and Alexanian, 1968; Axelsson and Hällén, 1972).

More recently, the incidence of M-components has been studied in the sera of normal healthy adult population and in different age groups (Hällén, 1963; Fine, Derycke and Boffa, 1965; Axelsson, Bachmann and Hällén, 1966; Axelsson and Hällén, 1972). During the last two decades M-components have been detected in the sera of patients suffering from malignant lymphoma (Azar, Hill and Osserman, 1957; Krauss and Sokal, 1966; Moore *et al.*, 1970), other forms of malignant disease (Osserman and Takatsuki, 1963; Hällén, 1966; Causey, 1967; Hosley, 1967; Migliore and Alexanian, 1968), and some non-malignant diseases (Owen, Pitney and O'Dea, 1959; Waldenström, 1964; Michaux and Heremans, 1969; Kanoh, 1970).

In view of the fact that M-components have been observed in patients with malignant diseases, it was decided to study their frequency in the sera of patients with histologically proven solid malignant neoplasms attending our Institute.

TABLE I.—*Survey of the Material*

| Type of neoplasm | Number of cases | Patients with M-components in the Serum: M-components in agar-electrophoresis M | F | Results of immunoelectrophoresis: "Paraprot" | Suspect | No abnormalities | Frequency (%) M + F |
|---|---|---|---|---|---|---|---|
| Ca of breast | 173 | — | 5 | — | 3 | 1+1* | 3 |
| Ca of lung | 172 | 22 | — | 2 | 8 | 10+2* | 13 |
| Ca of cervix | 130 | — | 7 | 1 | 4 | 2 | 5 |
| Ca of corpus uteri | 36 | — | 3 | — | 2 | 1 | 1 |
| Hodgkin's disease | 36 | — | 1 | — | — | 1 | 3 |
| Ca of bladder | 32 | 5 | 1 | 2 | 1 | 3 | 19 |
| Ca of larynx | 13 | 1 | — | — | — | 1 | — |
| Ca of colon | 11 | 1 | 1 | 1 | 1 | — | — |
| Ca of oesophagus | 11 | 2 | — | — | 1 | 1 | — |
| Lymphosarcoma | 9 | 1 | — | 1 | — | — | — |
| Ca of stomach | 7 | 1 | — | — | — | 1 | — |
| Ca of prostate | 7 | 2 | — | 1 | 1 | — | — |
| Ca of vagina | 5 | — | 1 | — | — | 1 | — |
| Ca of kidney | 4 | 1 | — | — | 1 | — | — |
| Combined tumour (seminoma–teratoma) | 2 | 1 | — | — | 1 | — | — |
| Multiple myeloma | 2 | 2 | — | 2 | — | — | — |
| Ca of fallopian tube | 1 | — | 1 | — | — | 1 | — |
| Waldenström's disease | 1 | 1 | — | 1 | — | — | — |
| Total | 652 | 40 | 20 | 11 | 23 | 23 | 44 |

Patients without M-components in the Serum

| Type of neoplasm | N | Type of neoplasm | N |
|---|---|---|---|
| Ca of ovary | 35 | Plasmocytoma (intra-osseus) | 2 |
| | | Ca of penis | 2 |
| Ca of rectum | 16 | Ewing's sarcoma | 2 |
| Reticulum cell sarcoma | 13 | Malignant teratoma of testis | 2 |
| Malignant melanoma | 11 | Mesothelioma of pleura | 2 |
| Seminoma | 8 | Osteosarcoma | 2 |
| Ca of salivary gland | 8 | Liposarcoma | 1 |
| Ca of vulva | 7 | Malignant sertoli cell tumour | 1 |
| Ca of pharynx | 6 | Paratesticular leiomyosarcoma | 1 |
| Ca of nasopharynx | 6 | Alveolar rhabdomyosarcoma | 1 |
| Ca of thyroid | 5 | Haemangioendothelioma | 1 |
| Ca of tongue | 5 | Malignant schwannoma | 1 |
| Ca of anus | 3 | Astrocytoma | 1 |
| Malignant lymphoma (unclassified) | 3 | Sarcoma of bladder | 1 |
| Leiomyosarcoma of the uterus | 2 | Endometrial stromal sarcoma | 1 |
| Ca of oral cavity | 2 | Carcinosarcoma of the body of the uterus | 1 |
| Ca of tonsil | 2 | Neuroblastoma | 1 |

* Immunoelectrophoretic results not available.

Total number of patients with and without M-components 807.

FIG. 1.—The three immunoelectrophoretic patterns observed in our patients with M-components in the serum. (a) a strong precipitation line, consistent with the presence of a monoclonal immunoglobulin of the IgG class; (b) a suspect pattern showing a split IgG precipitation line; (c) a normal pattern.

### MATERIAL AND METHODS

During a period of 13 months from March 1970 to April 1971 all new patients attending our Institute had a specimen of blood taken for electrophoretic examination. The population studied consisted of all new patients with proven malignant disease (both out-patients and in-patients), except for those with cutaneous neoplasms and leukaemia. At the same time all histological preparations relating to these patients were reviewed independently. When the histological material was found to be unsatisfactory and better preparations could not be obtained, the case was excluded from the study.

All sera were screened for the presence of M-components by agar gel electrophoresis using the method of Wieme (1959). Every specimen of serum which showed appearances suggesting the presence of an M-component was investigated further. This investigation consisted of immunoelectrophoresis of the serum using the method of Scheidegger (1955). At the same time the electrophoresis on agar gel was repeated, and the presence of an M-component in this specimen was a prerequisite for considering the serum as pôsitive. All M-components encountered were included.

The results showed that the sera examined exhibited 3 possible patterns, namely (1) a strong precipitation line, consistent with the presence of a monoclonal immunoglobulin (" paraprotein "); (2) a pattern which although it did not exhibit a definite " paraprotein " showed certain abnormalities, which were considered to be suspect; and (3) a pattern free from any abnormalities (Fig. 1).

### RESULTS

After exclusion of cases in which adequate histological material was not available the patient population under study consisted of 807 patients. There were 340 (43 %) males and 467 (57 %) females; the age and sex distribution of the patients is shown in Fig. 2. The histological diagnoses of the neoplasms found in the patients under study, the incidence of M-components, as well as the results of immunoelectrophoresis are shown in Table I.

FIG. 2.—Age and sex distribution of the patients.

TABLE II.—*Frequency of M-components in Different Age Groups*

| Age (years) | | 31–40 | 41–50 | 51–60 | 61–70 | 71–80 | 81–90 |
|---|---|---|---|---|---|---|---|
| Number of patients and frequency (%) | Male | — | 1 (3·8) | 6 (7·7) | 13 (12·0) | 16 (19·7) | 4 (33·3) |
| | Female | 1 (2·9) | 3 (3·6) | 8 (7·1) | 2 (1·7) | 3 (3·6) | 3 (11·5) |

Serum electrophoresis revealed the presence of M-components in 60 patients: 40 males and 20 females. The age distribution of these patients is shown in Fig. 2. The frequency of M-components was found to increase with age, and this was more evident in males (Table II).

Two patients with multiple myeloma and one patient with Waldenström's macroglobulinaemia diagnosed during the period under study were included. No other patient showed evidence of being affected by these diseases, and a special attempt was made to exclude this possibility in the patients with M-components in the serum.

Of the 60 patients with M-components 11, including 2 with multiple myeloma and one with Waldenström's macroglobulinaemia, were found to have a " paraprotein " (Fig. 1(a)). The typing of these immunoglobulins was as follows: 7 IgG (6 kappa and 1 lambda), 3 IgA and 1 IgM.

Twenty-four patients were found to have a " suspect " pattern (Fig. 1(b)). The typing of these immunoglobulins was as follows: 20 IgG, 3 IgA and 1 IgM. Twenty-two patients with M-components in the serum were found to have no abnormalities on immunoelectrophoresis (Fig. 1(c)). In all these patients the M-components were weak. In 3 patients with M-components, immunoelectrophoresis was not performed; in all 3 the M-component present formed a weak band.

M-components were found in the sera of patients with a wide variety of neoplasms (Table I). An increased frequency of M-components was found in patients with carcinomata of the lung and urinary bladder. Twenty-one patients whose sera contained an M-component were dead by the end of the period under study.

### DISCUSSION

This investigation was undertaken as a screening study in order to determine the frequency of M-components in the sera of patients with solid malignant neoplasms and to see whether there is a specific pattern. The patients attended a hospital concerned mainly with radiation therapy and the study may therefore suffer from a number of disadvantages associated with this type of investigation. The patient population studied is thus selected in the way that patients with neoplasms which are treated by radiotherapy predominate. In order to avoid any further selection, all patients with neoplastic disease attending the hospital during the period under study were included, except those with cutaneous neoplasms and leukaemia. This explains the inclusion of patients with multiple myeloma and Waldenström's macroglobulinaemia, diseases normally associated with the presence of M-components. A considerable number of patients with malignant lymphoma, a group of diseases which is known to be sometimes associated with the presence of M-components (Azar *et al.*, 1957; Krauss and Sokal, 1966; Moore *et al.*, 1970) were also included. In order to determine their significance all M-components observed in the sera of the patients studied were included. The frequency of M-components in the present study (7·0%) is very much higher than that observed by Migliore and Alexanian (1968), who found an incidence of 0·65% studying a similar population, except that patients with cutaneous neoplasms and a small number with benign neoplasms were included. Although this can be attributed partly to the inclusion of all M-components as detected by a sensitive technique, even the frequency of

" paraproteins " in the present study is higher (1·0 %).

If all the cases with immunoelectrophoretic abnormalities are included, the frequency is 4·0 %. Among the patients with M-components there was a male predominance of nearly 2 : 1 (1·85 : 1), although female patients made up 57 % of patients in the present study. There was a similar male predominance (1·75 : 1) among the patients with M-components studied by Migliore and Alexanian (1968), whose patient population was composed of 56 % female patients. In a study to determine the frequency of M-components in a normal adult population Axelsson *et al.* (1966) found a male predominance of 1·2 : 1 in a population consisting of 51 % females and a frequency of 0·9 %, using a less sensitive technique than that used in the present study or by Migliore and Alexanian (1968). Axelsson's results (1966) are thus higher than those of Migliore and Alexanian (1968) and are probably similar to those observed in the present study, as the frequency of M-components in their study is similar to the frequency of " paraproteins " in the present study.

Our results support the findings of Hällén (1963), Fine *et al.* (1965), Axelsson *et al.* (1966), and Fine, Lambin and Leroux (1972), who have reported that the incidence of M-components increases with age.

In the present study, in 22 out of 60 patients with M-components in the serum there were no abnormalities on immunoelectrophoresis. In all these cases the M-components were found to be weak, and there appears to be a relationship between the intensity of the M-component and the presence or absence of immunoelectrophoretic abnormalities. The M-components which were found were mostly of the IgG class, but a number of M-components of the IgA and one of the IgM class were also encountered, the patients with multiple myeloma and Waldenström's macroglobulinaemia having been excluded. These results are similar to the findings of Waldenström (1964), Axelsson *et al.* (1966), Hällén (1966) and Fine (1970). Migliore and Alexanian (1968) in their study did not encounter M-components of the IgA class, whereas the majority of the M-components found in patients with malignant neoplasms studied by Kanoh (1970) were of this class. This shows that there is no specific pattern in the immunoelectrophoretic results obtained by different investigators.

An important difference between the M-components encountered in multiple myeloma, Waldenström's macroglobulinaemia and some cases of malignant lymphoma, and those observed in patients with other neoplasms, non-neoplastic diseases and in the normal population is the intensity of the abnormal band and the concentration of the M-component in the serum. While in the former conditions the concentration of the M-component is usually relatively high and shows a gradual increase, in the latter it is usually low, producing a weak band and showing no significant rise when followed up over a long period of time (Migliore and Alexanian, 1968; Axelsson and Hällén, 1972). There appears to be no uniform or specific pattern as far as the location of the malignant neoplasms in patients with M-components is concerned, and neoplasms of many different organs have been encountered. Osserman and Takatsuki (1963) found that in their 31 patients the tumours were located mainly in the large intestine, nasopharynx and biliary tract. In the series reported by Migliore and Alexanian (1968) the most common sites were the breast, lungs, upper respiratory tract and large intestine. The number of patients with different neoplasms included in their study was not stated and therefore the actual frequency cannot be ascertained. Review of the literature failed to reveal any studies in which such information was given. In the present study, M-components were found in the sera of patients with neoplasms of many different organs. There was an increased frequency in patients with carcinoma of the lung and urinary bladder. This finding may be of interest, although its significance is not certain.

It has been stated by Hällén (1966) that the incidence of M-components in patients with malignant neoplasms, with the exception of multiple myeloma, Waldenström's macroglobulinaemia and malignant lymphoma, is not higher than in normal Swedish population. Migliore and Alexanian (1968) have stated that there is no evidence of an increased frequency of M-components in sera from patients with malignant neoplasms compared with a normal adult population.

Although the results of the present study indicate that the frequency of M-components in sera from patients with malignant neoplasms is higher than that found by Migliore and Alexanian (1968), there appears to be no evidence that it is higher than in a normal adult population.

### REFERENCES

Axelsson, U., Bachmann, R. & Hällén, J. (1966) Frequency of Pathological Proteins (M-Components) in 6,995 Sera from Adult Population. *Acta med. scand.*, **179**, 235.

Axelsson, U. & Hällén, J. (1972) A Population Study on Monoclonal Gammapathy. *Acta med. scand.* **191**, 111.

Azar, H. A., Hill, W. T. & Osserman, E. F. (1957) Malignant Lymphoma and Lymphatic Leukemia Associated with Myeloma Type Serum Proteins. *Am. J. Med.*, **23**, 239.

Causey, J. Q. (1967) IgG Paraproteinemia Associated with Bronchogenic Carcinoma. *Archs intern. Med.*, **119**, 509.

Fine, J. M. (1970) Study of the Frequency of Kappa and Lambda Light Chains in 347 Sera Containing a Monoclonal IgG, IgA, IgD, or Bence Jones Proteins. *Eur. J. clin. biol. Res.*, **15**, 199.

Fine, J. M., Derycke, C. & Boffa, G. A. (1965) Les dysglobulinémies monoclonales "essentielles". *Nouv. Revue fr. Hemat.* **5**, 729.

Fine, J. M., Lambin, P. & Leroux, P. (1972) Frequency of Monoclonal Gammapathy (M-components) in 13,400 Sera from Blood Donors. *Vox sang.*, **23**, 336.

Hällén, J. (1963) Frequency of "Abnormal" Serum Globulins (M-components) in the Aged. *Acta med. scand.*, **173**, 737.

Hällén, J. (1966) Discrete Gammaglobulin (M)-components in Serum. Clinical Study of 150 Subjects Without Myelomatosis. *Acta med. scand.*, **180**, Suppl. 462, 1.

Hosley, H. F. (1967) M-proteins, Plasmocytomas and Cancer. *Cancer, N.Y.*, **20**, 295.

Kanoh, T. (1970) The Behaviour of Immunoglobulin in Monoclonal Gammopathies and Their Classification and Pathogenesis. *Tohoku J. exp. Med.*, **102**, 369.

Krauss, S. & Sokal, J. E. (1966) Paraproteinemia in the Lymphomas. *Am. J. Med.*, **40**, 400.

Michaux, J. L. & Heremans, J. F. (1969) Thirty Cases of Monoclonal Immunoglobulin Disorders other than Myeloma and Macroglobulinemia. *Am. J. Med.*, **46**, 562.

Migliore, P. J. & Alexanian, R. (1968) Monoclonal Gammopathy in Human Neoplasia. *Cancer N.Y.*, **21**, 1127.

Moore, D. F., Migliore, P. J., Shullenberger, C. C. & Alexanian, R. (1970) Monoclonal Macroglobulinaemia in Malignant Lymphoma. *Ann. intern. Med.*, **72**, 43.

Osserman, E. F. & Takatsuki, K. (1963) Plasma Cell Myeloma: Gammaglobulin Synthesis and Structure. *Medicine, Baltimore*, **42**, 357.

Owen, J. A., Pitney, W. R. & O'Dea, J. F. (1959) "Myeloma" Serum Electrophoretic Patterns in Conditions Other than Myelomatosis. *J. clin. Path.*, **12**, 344.

Scheidegger, J. J. (1955) Une Micro-méthode de l'Immuno-électrophorèse. *Int. Archs Allergy appl. Immun.*, **7**, 103.

Waldenström, J. (1964) The Occurrence of Benign, Essential, Monoclonal (M-type), Non-macromolecular Hyperglobulinemia and its Differential Diagnosis. *Acta med. scand.*, **176**, 345.

Wieme, R. J. (1959) *Studies on Agar Gel Electrophoresis, Techniques, Applications.* Thesis, Brussels. Arscia.

Zawadzki, Z. A. & Edwards, G. A. (1967) Dysimmunoglobulinemia in the Absence of Clinical Features of Multiple Myeloma and Macroglobulinemia. *Am. J. Med.*, **42**, 67.